# Arterial Wave Reflection and Aortic Valve Stenosis: Diagnostic Challenges and Prognostic Significance

**DOI:** 10.3389/fcvm.2022.863968

**Published:** 2022-07-08

**Authors:** Stamatia Pagoulatou, Dionysios Adamopoulos, Georgios Rovas, Vasiliki Bikia, Hajo Müller, Georgios Giannakopoulos, Sarah Mauler-Wittwer, Marc-Joseph Licker, Nikolaos Stergiopulos, Stéphane Noble

**Affiliations:** ^1^Laboratory of Hemodynamics and Cardiovascular Technology, École Polytechnique Fédérale de Lausanne (EPFL), Lausanne, Switzerland; ^2^Faculty of Medicine, University of Geneva, Geneva, Switzerland; ^3^Department of Cardiology, Hôpitaux Universitaires de Genève (HUG), Geneva, Switzerland; ^4^Department of Anaesthesiology, Hôpitaux Universitaires de Genève (HUG), Geneva, Switzerland

**Keywords:** arterial wave reflection, aortic valve stenosis, arterial stiffness, transvalvular pressure gradients, arterial hypertension

## Abstract

**Introduction:**

Arterial wave reflection is an important component of the left ventricular afterload, affecting both pressure and flow to the aorta. The aim of the present study was to evaluate the impact of wave reflection on transvalvular pressure gradients (TPG), a key parameter for the evaluation of aortic valve stenosis (AS), as well as its prognostic significance in patients with AS undergoing a transcatheter aortic valve replacement (TAVR).

**Materials and Methods:**

The study population consisted of 351 patients with AS (mean age 84 ± 6 years, 43% males) who underwent a complete hemodynamic evaluation before the TAVR. The baseline assessment included right and left heart catheterization, transthoracic echocardiography, and a thorough evaluation of the left ventricular afterload by means of wave separation analysis. The cohort was divided into quartiles according to the transit time of the backward pressure wave (BWTT). Primary endpoint was all-cause mortality at 1 year.

**Results:**

Early arrival of the backward pressure wave was related to lower cardiac output (Q1: 3.7 ± 0.9 lt/min vs Q4: 4.4 ± 1.0 lt/min, *p* < 0.001) and higher aortic systolic blood pressure (Q1: 132 ± 26 mmHg vs Q4: 117 ± 26 mmHg, *p* < 0.001). TPG was significantly related to the BWTT, patients in the arrival group exhibiting the lowest TPG (mean TPG, Q1: 37.6 ± 12.7 mmHg vs Q4: 44.8 ± 14.7 mmHg, *p* = 0.005) for the same aortic valve area (AVA) (Q1: 0.58 ± 0.35 cm^2^ vs 0.61 ± 0.22 cm^2^, *p* = 0.303). In multivariate analysis, BWTT remained an independent determinant of mean TPG (beta 0.3, *p* = 0.002). Moreover, the prevalence of low-flow, low-gradient AS with preserved ejection fraction was higher in patients with early arterial reflection arrival (Q1: 33.3% vs Q4: 14.9%, *p* = 0.033). Finally, patients with early arrival of the reflected wave (Q1) exhibited higher all-cause mortality at 1 year after the TAVR (unadjusted HR: 2.33, 95% CI: 1.17–4.65, *p* = 0.016).

**Conclusion:**

Early reflected wave arrival to the aortic root is associated with poor prognosis and significant aortic hemodynamic alterations in patients undergoing a TAVR for AS. This is related to a significant decrease in TPG for a given AVA, leading to a possible underestimation of the AS severity.

## Introduction

Afterload is the mechanical load imposed on the left ventricle by both the aortic valve and the systemic circulation and is determined by complex time-varying phenomena. The arterial part has different components and it is best described by the propagative model of the human circulation, which consists of a distensible tube terminating at the peripheral resistance (1). The compliance of the tube permits the generation of a pressure wave, that travels along the arterial tree from the aortic root to the periphery (2).

From a physiological standpoint, the best fitting propagative model also predicts the presence of retrograde pressure waves moving throughout the arterial tree in the opposite direction (from the periphery to the aortic root) (1). This model consist the basis of the arterial reflection theory, which describes arterial reflections occuring at the elastic tube’s end, a theoretical area characterized by high levels of resistance (2).

**FIGURE 1:**
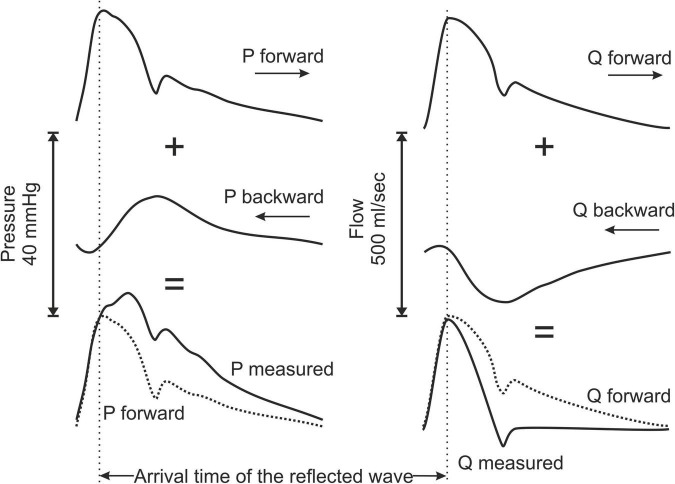
Schematic representation describing the principle of the arterial wave reflection theory and the effects on pressure and flow contour waveform by the reflected waves. P, pressure; Q, blood flow.

The physiological implications of this phenomenon have been extensively studied, especially as a key mechanism for the development of arterial hypertension with advancing age (3). One of the crucial factors is the timing of the arrival of the reflected wave and specifically how it relates to the ejection period. Many factors have been shown to influence this parameter such as the distance of the reflection sites, the tone of the arterioles as well as the velocity at which the waves travel along the arterial tree, which is determined by the compliance of the system (2). In case of early return (before the closure of the aortic valve), the reflected pressure wave adds to the pressure burden imposed to the left ventricle, becomes a considerable part of the afterload and decelerates blood flow (Figure 1). This mechanism provides the pathophysiological background that explains the prognostic impact (cardiovascular events and mortality) of early wave reflections in different populations such as patients with arterial hypertension, end-stage renal and coronary artery disease (4–7).

Aortic valve stenosis (AS) is the most common valvular heart disease in the Western world impacting significantly morbidity and mortality especially in the elderly population (8). In the clinical setting, the evaluation of AS severity relies predominantly on the transvalvular pressure gradients (TPG), defined as the difference in pressure between the left ventricle and the aortic root. A mean TPG of equal or more than 40 mmHg, typically measured by Doppler echocardiography, suggests a severe AS and represents a class I indication for aortic valve replacement in symptomatic patients (9).

Since TPG depends on the transvalvular blood flow (9–12), and based on the arterial wave reflection theory, we hypothesized that TPG may be influenced by the incidence of wave reflection to the aorta. In order to test this hypothesis, we explored the associations between TPG and arterial wave reflection indices in patients with AS, who underwent a thorough hemodynamic assessment before transcatheter aortic valve replacement (TAVR). Finally, in the same population, we explored the prognostic significance of early wave reflection by assessing all-cause mortality at 1 year.

## Materials and Methods

### Study Population

**FIGURE 2:**
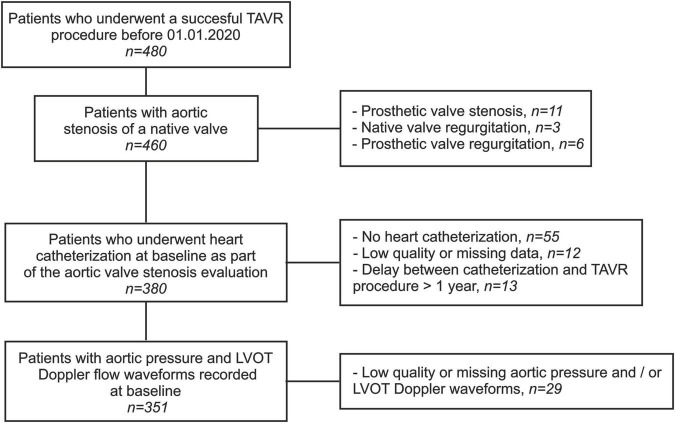
Study flowchart. TAVR, transcatheter aortic valve replacement; LVOT, left ventricular outflow tract.

This is a retrospective study based on data collected from the medical records of all patients who underwent a successful TAVR in our department from June 2008 to December 2019 (*n* = 480). The study population comprised patients referred for symptomatic AS of a native valve while presenting a high or intermediate risk for a conventional surgical approach. Sixty-seven patients were excluded due to unavailable, low quality, or missing data from baseline heart catheterization. Twenty-nine patients were excluded due to missing or low-quality baseline echocardiographic Pulsed Wave Doppler measurements and/or invasive aortic pressure measurements during the TAVR. Finally, thirteen patients were excluded because of a delay exceeding 1 year between the baseline heart catheterization and the TAVR. The final cohort consisted of 351 patients, and all data were anonymized prior to analysis. Informed written consent was obtained from each patient for inclusion in the local TAVR database as part of the Swiss prospective registry (NCT1368250) approved by the local Ethics Committee. A detailed study flowchart is depicted in Figure 2.

The study population was divided into four groups corresponding to the quartiles (Q) of the transit time of the backward pressure wave (BWTT). For the purpose of the current study, meaningful comparisons in BWTT between subjects can only be performed after taking into account for the relative timing of the arrival of the reflected wave to the systolic period. For this reason, values are expressed as percentage of the ejection duration (ED): Quartile 1 (Q1), *n* = 87, BWTT ≤ 11.8% of the ED; Quartile 2 (Q2), *n* = 88, BWTT from 11.9 to 15.5% of the ED; Quartile 3 (Q3), *n* = 88, BWTT from 15.6 to 22.3% of the ED and; Quartile 4 (Q4), *n* = 88, BWTT ≥ 22.4% of the ED.

### Right and Left Heart Catheterization

All patients underwent a baseline heart catheterization as part of the standard evaluation of the AS. During this examination, cardiac output (CO) was measured for all patients either by the thermodilution or the modified Fick method with estimated oxygen consumption. CO was also indexed to body surface area (BSA) and cardiac index (CI) was calculated. Stroke volume (SV) was calculated as the ratio of the CO to the heart rate (HR) and was indexed to BSA (SV index [SVi]). Pulmonary artery and wedge pressures were also obtained, while a diagnostic coronary angiography was performed on all patients.

On the day of the TAVR, invasive recordings of the baseline pressure waveform in the aortic root were acquired. For all but five patients, simultaneous left ventricular pressure measurements were available. The heart catheterization protocol included a first 6F “pigtail” catheter (Cordis), which was advanced through the stenotic aortic valve into the left ventricle from the vascular access for the transcatheter prosthetic valve and a second 6F “pigtail” catheter which was advanced to the aortic root using a second vascular access. Both catheters were connected to a pressure line and a calibrated transducer. In some patients, a double lumen catheter (Langston) was used. The pressure curves were simultaneously recorded over several heartbeats and were subsequently analyzed offline.

### Echocardiography

A complete transthoracic echocardiography (TTE) in supine position was performed prior to the TAVR in all study participants. All measurements were conducted by an experienced cardiologist according to standard recommendations for TTE (13). Acquired images were transferred to a dedicated workstation for subsequent offline analysis (IntelliSpace Cardiovascular 5.1, Philips Medical Systems Nederland B.V.). Data on left ventricular geometry were collected, and left ventricular mass was calculated according to the Devereux formula (14). The proximal velocity profile was acquired in the left ventricle outflow tract via Pulsed Wave Doppler in the standard apical 5-chamber view. The aortic flow waveform was subsequently derived after calibration for the invasively measured SV. Aortic valve TPG, ejection fraction (EF), aortic valve area (AVA), AVA indexed to BSA (AVAi), and qualitative evaluation of other valve abnormalities (mitral, tricuspid) were extracted from the standard echocardiographic reports.

### Aortic Stenosis Classification

AS classification was performed by the application of the diagnostic criteria proposed by the European Society of Cardiology Guidelines for the management of valvular disease (15); (i) High-gradient AS: AVA < 1 cm^2^ or AVAi < 0.6 cm^2^/m^2^ and mean TPG ≥ 40 mmHg; (ii) Low-gradient AS with reduced EF: AVA < 1 cm^2^ or AVAi < 0.6 cm^2^/m^2^, mean TPG < 40 mmHg and EF < 50%; (iii) Low-flow, low-gradient AS with preserved EF: AVA < 1 cm^2^ or AVAi < 0.6 cm^2^/m^2^, mean TPG < 40 mmHg, EF ≥ 50% and SVi ≤ 35 ml/m^2^; (iv) Normal-flow, low-gradient AS with preserved EF: AVA < 1 cm^2^ or AVAi < 0.6 cm^2^/m^2^, mean TPG < 40 mmHg, EF ≥ 50% and SVi > 35 ml/m^2^.

### Wave Separation Analysis

The left ventricle and aortic root pressure curves recorded before the TAVR were digitized for each patient. A custom, in-house Matlab code was developed to identify the beginning and end of each heartbeat automatically, and the average pressure curves over several (4–8) heartbeats were computed. Subsequently, pressure waveform analysis was performed, and key features were determined, including (i) left ventricle systolic and end-diastolic pressures, (ii) the invasive TPG, calculated as the difference between the left ventricle and aortic pressures (area under the curve, peak to peak and mean), (iii) the aortic systolic, diastolic, mean and pulse pressures and (iv) the valvulo-arterial impedance, defined as the ratio of systolic left ventricular pressure over SVi. According to the Gorlin formula (12), the AVA and AVAi were calculated as the ratio of mean flow and mean TPG:


(1) $$A\,V\,A=\frac{Q_{m\,e\,a\,n}}{44.3\,\sqrt{T\,P\,G_{m\,e\,a\,n}}}$$

The invasive aortic pressures were subsequently combined with the TTE flow curves for wave separation analysis. The two curves were synchronized for each patient by adopting the second derivative approach, whereby the time lag between the two signals was corrected by calculating the maxima of the second time derivatives (16). Note that any difference in HR between the pressure and flow measurements was accounted for by truncating or extending the diastolic portion of the flow wave. Subsequently, wave separation analysis was performed by applying the standard methodology in the frequency domain. More specifically, the input impedance was derived from the synchronized pressure and flow curves as the ratio of the corresponding harmonics. Aortic characteristic impedance (Zc) was then identified by averaging the input impedance modulus of the 3rd to 9th harmonics (after excluding outlier values greater than three times the median value of input impedance modulus over that range of harmonics). The forward and backward pressure and wave components were subsequently calculated as described by Westerhof et al. (17):


(2) $$P_{f\,o\,r\,w\,a\,r\,d}=\frac{P+Z_{c}\,Q}{2}\,a\,n\,d\,P_{b\,a\,c\,k\,w\,a\,r\,d}=\frac{P-Z_{c}\,Q}{2}$$

(3) $$Q_{f\,o\,r\,w\,a\,r\,d}=\frac{P_{f\,o\,r\,w\,a\,r\,d}}{Z_{c}}\,a\,n\,d\,Q_{b\,a\,c\,k\,w\,a\,r\,d}=-\frac{P_{b\,a\,c\,k\,w\,a\,r\,d}}{Z_{c}}$$

Key features of the forward and backward pressure waves were identified, including the magnitude and timing of the peak pressure, the wave amplitude, and the BWTT, identified by the foot of the curve. Finally, the reflection coefficient was evaluated as the ratio of the backward wave to the forward wave amplitudes. The synchronized pressure and flow signals were additionally used for the calculation of the equivalent total vascular resistance (TVR) and total arterial compliance (TAC) via parameter-fitting on a 2-element Windkessel model as described by Stergiopulos et al. (18).

### Procedure Characteristics

Aortic valve replacement was performed by the use of the Medtronic self-expanding CoreValve and Evolut devices (Medtronic Inc., Minneapolis, MN, United States, *n* = 341, 97.1%), the Edwards Sapien S3 (Edwards Lifesciences SA, CA, United-States, *n* = 28, 7.9%) or the Boston neo Accurate (Boston Scientific AG, MA, United States, *n* = 5, 1.5%). Device implantation success was systematically evaluated for all interventions according to the Valve Academic Research Consortium-2 consensus Document criteria (19).

### Follow-Up

A post-TAVR follow-up was performed for all patients at 1-, 6-, and 12-months intervals through a clinical visit. All baseline clinical characteristics and procedural and follow-up data were stored in a dedicated database using a secured online platform^1^ (OpenClinica LLC, Waltham, MA, United States). The primary study endpoint was all-cause mortality at 1 year. Events were adjudicated by an external clinical committee.

### Statistical Analysis

Categorical variables are reported as counts with percentages. Continuous variables are expressed as mean and standard deviation or as the median and interquartile range for variables with non-normal distribution (normality was assessed by visual inspection of the frequency distributions). Categorical variables are compared among groups by the use of Pearson Chi-Square or the Fischer exact test as appropriate. Continuous variables were compared among the groups using analysis of variance (ANOVA) or the Kruskal-Wallis test for the non-normally distributed data. Levene’s test was used to assess the homogeneity of variance among the compared groups, and in case of violation, Welch’s ANOVA test was used. In order to assess the independent effect of the BWTT on TPG multiple linear regression model analysis was performed treating BWTT as a continuous variable and after adjusting for the following parameters: Aortic systolic blood pressure, AVA (estimated by the Gorlin formula), Zc, TVR, TAC, gender and height. One-year all-cause mortality rates was calculated from Kaplan-Meier analysis for patients presenting the earliest return of the reflected wave (Q1) as compared to the rest of the population (Q2–Q4). Cox-regression analysis for the same groups was performed to compute hazard ratios and the 95% confidence intervals after verification of the proportional hazard assumption. A multivariate Cox-regression model was used in order to adjust comparisons between the two groups for potential confounding mortality factors (Model A: STS Score and gender, Model B STS score, gender and tricuspid regurgitation, Model C: Device success). Statistical significance was assumed at a 2-sided *P*-value level of 0.05. Statistical analysis was performed in IBM SPSS statistics (IBM Corp. Released 2020. IBM SPSS Statistics for Windows, Version 27.0. Armonk, NY: IBM Corp).

## Results

### Baseline Characteristics

The baseline characteristics of the study population are presented in Table 1. Baseline characteristics did not differ among the 4 groups, except for low height and female gender that were associated with early arrival of the reflected wave (*p* = 0.009 and 0.002, respectively).

**TABLE 1:** Demographic and clinical characteristics of the study population according to the BWTT quartiles.

	Backward wave transit time				P-value
		
	Q1 (*n = 87*)	Q2 (*n = 88*)	Q3 (*n = 88*)	Q4 (*n = 88*)	
**Demographics**					

Age (years)	84 ± 6	85 ± 6	83 ± 6	82 ± 6	0.071
Height (cm)	164 ± 8	162 ± 8	166 ± 10	166 ± 10	0.009
Weight (Kg)	69 ± 14	71 ± 15	70 ± 15	75 ± 15	0.089
BMI (kg/m^2^)	25.8 ± 5.4	26.8 ± 5.0	25.4 ± 4.6	27.0 ± 4.9	0.084
BSA (m^2^)	1.76 ± 0.19	1.78 ± 0.21	1.8 ± 0.23	1.85 ± 0.21	0.058
Gender (males, *n*, %)	29 (33)	29 (33)	43 (50)	50 (57)	0.002

**Pre-intervention risk scores**					

Euroscore (%, *n* = 343)	13.6 [8.9–22.1]	15.2 [10.1–23.3]	13.5 [9.4–20.9]	13 [8.9–18.3]	0.336
STS Score (%, *n* = 343)	5.4 [3.4–8.2]	5.3 [3.7–8.3]	4.9 [3.1–7.3]	4.1 [2.8–5.8]	0.013

**Comorbidities and risk factors**					

Diabetes (%)	27 (31)	30 (34)	22 (25)	24 (27)	0.560
Dyslipidaemia (%)	62 (71)	66 (75)	56 (64)	58 (66)	0.353
Arterial hypertension (%)	69 (79)	73 (83)	70 (80)	72 (82)	0.911
Smokers (%)	8 (9)	3 (3)	7 (8)	8 (9)	0.095
CAD (%)	49 (56)	45 (51)	51 (58)	44 (50)	0.660
Previous MI (%)	10 (12)	13 (15)	9 (10)	12 (14)	0.798
PAD (%)	12 (14)	13 (15)	15 (17)	7 (8)	0.332
COPD (%)	17 (20)	13 (15)	14 (16)	15 (17)	0.854
Renal failure (%)	40 (51)	47 (53)	43 (49)	46 (52)	0.937
Cancer (%)	15 (17)	21 (24)	17 (19)	24 (27)	0.373
Atrial fibrillation/flutter (%)	33 (38)	26 (30)	27 (31)	29 (33)	0.650

**Presence of symptoms**					

NYHA III or IV (%)	65 (75)	66 (75)	61 (69)	64 (73)	0.822
Syncope (%, *n* = 342)	5 (6)	11 (13)	11 (13)	13 (16)	0.249
Angina (%)	22 (25)	14 (16)	22 (25)	10 (12)	0.080

**Baseline medications**					

Aspirin (%)	52 (60)	53 (60)	47 (53)	42 (48)	0.291
Oral anticoagulation (%)	27 (31)	26 (30)	25 (28)	25 (28)	0.978
Beta-blockers (%)	35 (40)	38 (43)	33 (38)	34 (39)	0.880
ACE inhibitors (%)	23 (26)	15 (17)	20 (23)	21 (24)	0.499
ARBs (%)	35 (40)	38 (43)	33 (38)	34 (39)	0.880
Statin (%)	52 (60)	50 (57)	44 (50)	49 (56)	0.619

*Continuous variables expressed as mean ± standard deviation or median and interquartile range. Categorical variables are expressed in absolute counts and (percentages). P-values obtained by ANOVA or Chi-Square test.*

*BMI, body mass index; BSA, body surface area; STS, Society of Thoracic Surgeons; CAD, coronary artery disease; PAD, peripheral artery disease; COPD, chronic obstructive pulmonary disease; NYHA, New York Heart Association; ACE, angiotensin-converting enzyme; ARBs, angiotensin receptor blockers.*

### Invasive Hemodynamics

**FIGURE 3:**
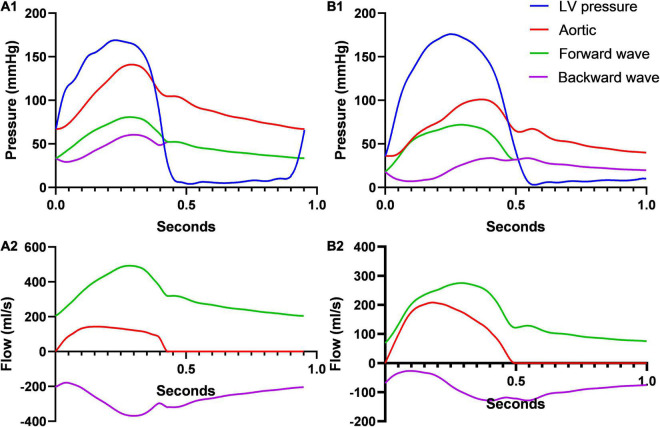
Pressure and flow wave separation analysis for two study participants from the Q1 **(A)** and the Q4 **(B)** groups. Pressure and flow wave separation analysis in a patient with an early (transit time 0.041 s, reflection magnitude 66%, panels **A1,A2**) and a late reflection wave arrival (transit time 0.167 s, reflection magnitude 49%, **B1,B2**) with the same AVA (0.55 cm^2^). Early reflection arrival is associated with a much more prominent deceleration of the aortic flow due to the backward wave and a decreased SV (**A2** vs. **B2**, 47 ml vs 68 ml accordingly). In addition, early arrival is associated with increased aortic pressure during systole, with left ventricular pressure being comparable between the two patients (**A1** vs. **B1**).

No significant differences were noted among groups in terms of HR, ED, left ventricular systolic, end-diastolic, mean pulmonary artery, and wedge pressures (Table 2). Early arrival of the reflected wave was associated with decreased aortic flow as assessed by CO, CI, SV, SVi, and mean flow rate. Likewise, early arrival of the reflected wave was associated with higher pressure-derived parameters (aortic systolic, mean, and pulse pressures). Finally, early arrival of the reflected wave was associated with lower invasive TPG expressed as area under the curve (*p* = 0.027), peak to peak (*p* = 0.012), and mean (*p* = 0.011) gradients, while AVA remained constant among groups. Two characteristic cases from the Q1 and Q4 groups are depicted in Figure 3.

**TABLE 2:** Invasive and echocardiographic parameters according to the BWTT quartiles.

	Backward wave transit time				*P*-*value*
	
	Q1 (*n = 87*)	Q2 (*n = 88*)	Q3 (*n = 88*)	Q4 (*n = 88*)	
**Invasive hemodynamics**					

Heart rate (bpm)	76 ± 14	75 ± 12	76 ± 12	75 ± 14	0.916
Ejection duration (ms)	414 ± 55	408 ± 50	417 ± 53	401 ± 51	0.184
Cardiac output (lt/min)	3.7 ± 0.9	4.0 ± 1.0	3.9 ± 1.0	4.4 ± 1.0	<0.001
Cardiac index (lt/min/m^2^)	2.1 ± 0.5	2.2 ± 0.5	2.2 ± 0.5	2.4 ± 0.5	0.003
Cardiac output normalized for height (lt/min/m)	2.3 ± 0.5	2.4 ± 0.6	2.4 ± 0.6	2.6 ± 0.6	<0.001
Stroke volume (ml)	50.4 ± 14.8	54.4 ± 17.3	53.6 ± 17.1	61.1 ± 19.1	0.001
Stroke volume index (ml/m^2^)	28.4 ± 7.6	30.4 ± 8.1	29.6 ± 7.8	32.7 ± 8.5	0.004
Stroke volume normalized for height (ml/m^3^)	30.7 ± 8.7	33.4 ± 9.9	32.1 ± 9.6	36.6 ± 10.7	0.001
Mean flow rate (ml/s)	123 ± 37	135 ± 45	129 ± 43	153 ± 47	<0.001
LV max pressure (mmHg, *n* = 346)	171 ± 29	168 ± 29	166 ± 32	165 ± 30	0.630
LV end-diastolic pressure (mmHg, *n* = 346)	18 ± 7	16 ± 8	17 ± 7	17 ± 9	0.694
Aortic SBP (mmHg)	132 ± 26	123 ± 25	122 ± 25	117 ± 26	0.001
Aortic MBP (mmHg)	85 ± 15	76 ± 15	80 ± 16	78 ± 16	0.009
Aortic DBP (mmHg)	55 ± 11	51 ± 11	54 ± 12	53 ± 12	0.089
Aortic PP (mmHg)	77 ± 22	72 ± 21	68 ± 23	63 ± 22	<0.001
Ventricular-aortic pressure gradient (AUC, mmHg*s, *n* = 343)	11.8 ± 5.7	13.8 ± 6.4	14.5 ± 6.7	14.3 ± 6.7	0.027
Ventricular-aortic pressure gradient (peak, mmHg, *n* = 346)	38 ± 17	45 ± 22	45 ± 21	48 ± 22	0.012
Ventricular-aortic pressure gradient (mean, mmHg, *n* = 343)	28 ± 13	34 ± 15	35 ± 15	35 ± 15	0.011
Zva (mmHg/ml/m^2^, *n* = 345)	6.4 ± 2.1	5.9 ± 2.1	6.0 ± 1.9	5.3 ± 1.6	0.006
AVA (Gorlin, cm^2^, *n* = 341)	0.58 ± 0.35	0.56 ± 0.21	0.54 ± 0.21	0.61 ± 0.22	0.303
PAP mean (mmHg)	26 ± 10	25 ± 9	27 ± 11	26 ± 11	0.466
Wedge pressure (mmHg)	15 ± 8	14 ± 7	15 ± 8	15 ± 8	0.296

**Echocardiographic parameters**					

Transvalvular max pressure gradient (mmHg)	63.9 ± 20.1	68. ± 19.4	71.7 ± 24.2	75.9 ± 22.9	0.003
Transvalvular mean pressure gradient (mmHg)	37.6 ± 12.7	40.2 ± 12.6	42.4 ± 14.7	44.8 ± 14.7	0.005
Transvalvular max velocity (cm/s)	395 ± 64	410 ± 60	418 ± 72	430 ± 65	0.005
Ejection duration (ms)	343 ± 45	337 ± 47	345 ± 47	330 ± 44	0.123
Ejection fraction (%)	62.5 [50–65]	62.5 [50–65]	61.3 [48.8–62.5]	62.5 [53.1–64.4]	0.485
AVA (continuity equation, cm^2^)	0.75 ± 0.21	0.75 ± 0.2	0.70 ± 0.21	0.72 ± 0.17	0.255
LV mass (g, *n* = 346)	189 ± 59	200 ± 67	206 ± 71	231 ± 70	<0.001
End diastolic LV diameter (cm)	4.6 ± 0.8	4.4 ± 0.8	4.7 ± 0.7	4.8 ± 0.7	0.041
Aortic regurgitation (≥moderate)	3 (3.4)	2 (2.3)	3 (3.4)	6 (6.8)	0.447
Mitral regurgitation (≥moderate)	7 (8)	3 (3.4)	2 (2.3)	8 (9.1)	0.135
Tricuspid regurgitation (≥moderate)	11 (12.6)	2 (2.3)	4 (4.5)	5 (5.7)	0.031

*Continuous variables expressed as mean ± standard deviation or median and interquartile range. Categorical variables are expressed in absolute counts and (percentages). P-values obtained by ANOVA or Chi-Square test.*

*LV, left ventricle; SBP, systolic blood pressure; DBP, diastolic blood pressure; MBP, mean blood pressure; PP, pulse pressure; AUC, area under the curve; Zva, valvulo-arterial impedance; AVA, aortic valve area; PAP m, mean pulmonary artery pressure; BSA, body surface area.*

### Echocardiographic Parameters

**FIGURE 4:**
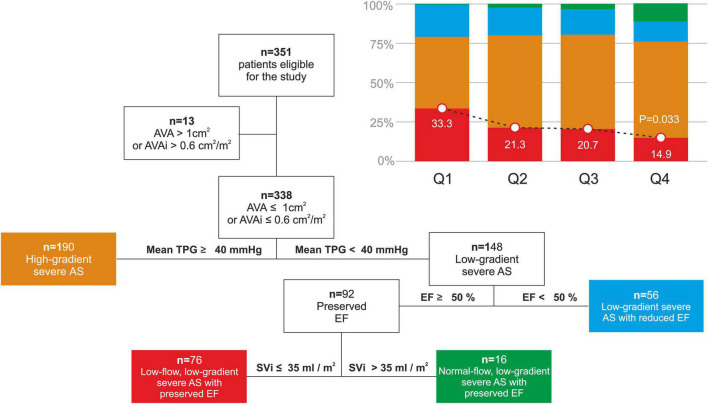
Aortic stenosis classification and incidence of the low-flow, low-gradient severe AS with preserved EF according to the BWTT quartiles. AS, aortic stenosis; AVA, aortic valve area; AVAi, aortic valve area indexed for body surface area; EF, ejection fraction; TPG, transvalvular pressure gradient; SVi, stroke volume indexed for body surface area.

Lower Doppler-derived mean (*p* = 0.005), maximum TPG (*p* = 0.003), and maximum transvalvular velocity (*p* = 0.005) were all associated with shorter BWTT (Table 2). AVA calculated according to the continuity equation was comparable among groups. No difference in EF was noted among groups. Tricuspid regurgitation was associated with early arrival of the reflected wave (Table 2, *p* = 0.031), as well as the prevalence of the low-flow, low-gradient AS with preserved EF (Q1: 33.3% vs Q2: 21.3% vs Q3: 20.7% vs Q4: 14.9%, *p* = 0.033, Figure 4).

### Arterial Tree and Wave Separation Analysis

Early arrival of the backward wave was associated with higher TVR (*p* < 0.001), lower TAC (*p* = 0.041) and lower Zc (*p* = 0.002, Table 3). No difference among groups was noted in the forward wave amplitude and timings. Backward wave amplitude and the reflection coefficient were all associated with an early arrival of the reflected wave (*p* < 0.001 for all).

**TABLE 3:** Characteristics of the arterial tree and of the forward and backward pressure waveforms as derived from the wave separation analysis according to the BWTT quartiles.

	Backward wave transit time				*P*-*value*
		
	Q1 (*n = 87*)	Q2 (*n = 88*)	Q3 (*n = 88*)	Q4 (*n = 88*)	
**Arterial tree**					

Systemic vascular resistance (mmHg/ml)	1.65 ± 0.64	1.45 ± 0.54	1.50 ± 0.47	1.27 ± 0.46	<0.001
Total arterial compliance (ml⋅ mmHg^–1^)	0.49 ± 0.25	0.58 ± 0.31	0.58 ± 0.28	0.61 ± 0.25	0.041
Characteristic impedance of the aorta (mmHg/ml)	0.14 ± 0.06	0.17 ± 0.08	0.18 ± 0.08	0.18 ± 0.07	0.002

**Wave separation analysis**					

Forward wave amplitude (mmHg)	49 ± 13	49 ± 15	48 ± 16	48 ± 16	0.841
Time to forward wave peak (ms)	258 ± 43	250 ± 40	258 ± 46	251 ± 44	0.465
Backward wave amplitude (mmHg)	33 ± 11	28 ± 9	27 ± 10	25 ± 9	<0.001
Time to backward wave peak (ms)	294 ± 47	305 ± 51	329 ± 65	354 ± 62	<0.001
Backward wave transit time (ms)	40 ± 9	55 ± 8	77 ± 11	117 ± 23	<0.001
Reflection coefficient (%)	66 ± 13	59 ± 10	58 ± 12	53 ± 10	<0.001

*Continuous variables expressed as mean ± standard deviation. P-values obtained by ANOVA.*

### Multiple Linear Regression Analysis

**FIGURE 5:**
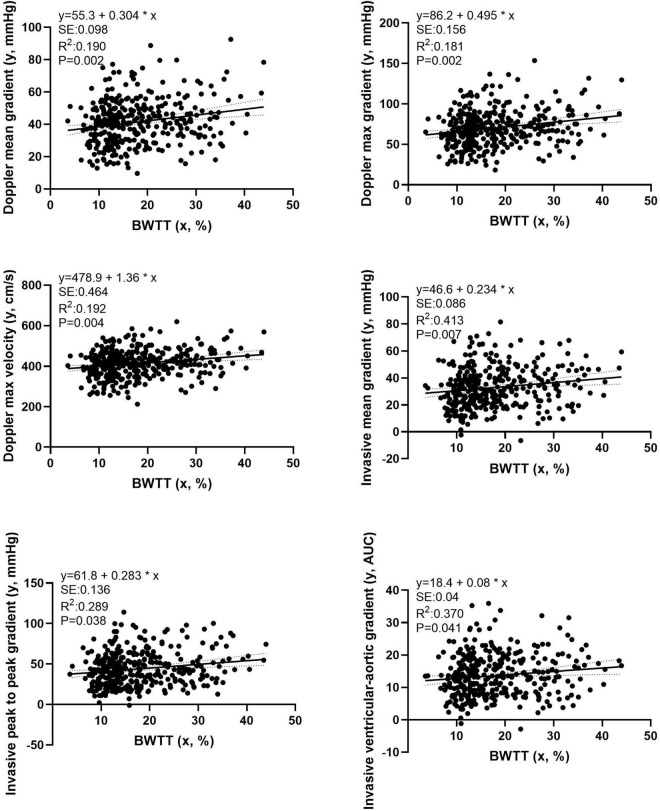
Multiple linear regression analysis examining the independent effect of the BWTT on transvalvular pressure gradients obtained by either echocardiographic or invasive evaluation. Independent variables: Aortic systolic blood pressure; Aortic valve area (estimated by the Gorlin formula); Aortic characteristic impedance; Systemic vascular resistance; Total arterial compliance; gender and height.

In multivariate analysis, BWTT remained a strong, independent predictor of all TPG measures after adjusting for gender and height, Zc, TVR, and TAC (Figure 5, *p* < 0.05 for all).

### Transcatheter Aortic Valve Replacement Intervention

Data on the TAVR procedure are presented in Table 4. Trans-femoral access was the most used approach (*n* = 341), followed by sub-clavian (*n* = 4) and trans-apical access (*n* = 2). 44 patients (12.5%) underwent a concomitant procedure (coronary angioplasty). Device success was achieved in 325 interventions (92.5%), which was comparable among the groups (Table 4).

**TABLE 4:** Transcatheter aortic valve replacement (TAVR) procedural characteristics.

	Backward wave transit time				P-value
		
	Q1 (*n = 87*)	Q2 (*n = 88*)	Q3 (*n = 88*)	Q4 (*n = 88*)	
**Access site**					0.764
Trans-femoral (%)	84 (96.6)	86 (97.7)	85 (96.6)	86 (97.7)	
Trans-apical (%)	0 (0)	1 (1.1)	1 (1.1)	0 (0)	
Sub-clavian (%)	1 (1.1)	1 (1.1)	2 (2.3)	2 (2.3)	
Other (%)	2 (2.3)	0 (0)	0 (0)	0 (0)	
**Prosthetic valve type**					0.827
Medtronic CoreValve (%)	79 (89.7)	79 (89.8)	79 (89.8)	83 (94.3)	
Edwards Sapien (%)	8 (9.2)	8 (8.0)	7 (8.0)	5 (5.7)	
Boston Acurate (%)	1 (1.1)	2 (2.3)	2 (2.3)	0 (0)	
**Procedural specifications**					
Concomitant procedure (%)	13 (14.9)	9 (10.2)	13 (14.8)	9 (10.2)	0.642
Device success (%)	79 (90.8)	86 (97.7)	82 (93.2)	78 (86.6)	0.112

### Clinical Outcomes

**FIGURE 6:**
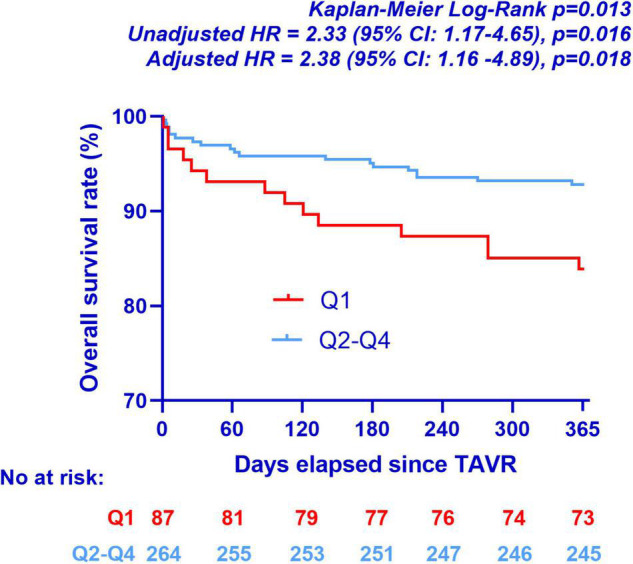
Kaplan-Meier curves for all-cause mortality at 1 year according to BWTT quartiles. Model A: adjusted HR = 2.38 (95% CI: 1.16–4.89, *p* = 0.018), covariates: gender and STS Score. Model B: adjusted HR = 2.00 (95% CI: 0.95–4.24, *p* = 0.064), covariates: gender, STS Score and tricuspid regurgitation. Model C: adjusted HR = 2.24 (95% CI: 1.12–4.47, *p* = 0.022), covariates: Device success. TAVR: transcatheter aortic valve replacement.

Clinical follow-up was completed for the totality of the study population. Figure 6 presents mortality data stratified according to BWTT. Patients with early backward wave return (Q1, ≤25th percentile) exhibited higher all-cause mortality rates at 1 year (unadjusted HR 2.33; 95% CI: 1.17–4.65, *p* = 0.016), as compared to the rest of the study population. This remained significant even after adjustment for baseline differences including gender and STS score (Model A; adjusted HR 2.38; 95% CI: 1.16–4.89, *p* = 0.018), for device success rate (Model C: adjusted HR = 2.24 (95% CI: 1.12–4.47, *p* = 0.022), but not after adjustment for tricuspid regurgitation which was different between the groups (Model B, adjusted HR 2.00, 95% CI: 0.95–4.24, *p* = 0.064).

## Discussion

The main findings of the present study may be summarized as follows: In patients with AS, the early arrival of the wave reflection to the aorta is associated with (a) lower aortic TPG (as assessed either by Doppler echocardiography or heart catheterization) for the same AVA; (b) lower CO, lower SV, and lower mean flow rate during systole, (c) higher aortic systolic and mean pressures and finally (d) poor prognosis as assessed by all-cause 1 year mortality.

Although a causal relationship cannot be established, the present study provides evidence that wave reflections may influence TPG measurements possibly through a flow-dependent mechanism affecting the accuracy of AS evaluation. The shift towards earlier arrival of the reflected waves increases pressure during systole and decelerates substantially the aortic transvalvular flow. According to the Gorlin formula, for a given AVA, TPG depends exclusively on the transvalvular flow, which explains the observed associations (12).

Notably, the present findings suggest a possible physiological explanation for the discrepancies observed in patients with low-flow, low-gradient severe AS and preserved EF. Since its introduction in 2007, this entity has remained a great challenge for the clinical cardiologist both in terms of diagnosis and treatment. It is characterized by a very small AVA (less or equal to 1 cm^2^) corresponding to a severe AS, but with a mean TPG below 40 mmHg, classifying the stenosis as less severe. The low TPG in these patients is explained by a low-flow state, which is defined by a SVi ≤ 35 ml/m^2^. The “paradox,” though, lies in the fact that the SV is low while at the same time the EF is preserved (≥50%) (20). Different factors have been incriminated for this low-flow state, including atrial fibrillation, small left ventricular cavity size, impaired diastolic filling, left restrictive ventricular physiology, and concomitant valvopathies (21). The findings of the present study suggest that an enhanced arterial wave reflection may also participate in the pathogenesis of the low-flow state in these patients. This is further supported by the fact that reduced TAC (a significant determinant of wave propagation velocity and thus wave reflection) has been consistently observed in patients with low-flow, low-gradient severe AS with preserved EF (20, 22).

In our study, enhanced reflections were associated with stiffer arterial trees (low TAC), which, however, had lower Zc, i.e., lower proximal aortic stiffness. In young adults, the proximal aorta is highly compliant, whereas the peripheral arteries are relatively stiff. In terms of wave propagation, this suggests an important impedance mismatch between the compliant aorta and the branch vessels, which generates reflections (23). Disproportionate stiffening of the proximal aorta and augmentation of the characteristic impedance (typically observed during aging) are therefore associated with a decrease in the impedance mismatch and a decrease in the backward wave amplitude (24). Following this paradigm, we may infer that the enhanced wave reflections in the Q1 group are likely due to a more pronounced impedance mismatch between central and peripheral arteries.

It is interesting to note that the decrease in SV observed with enhanced wave reflection was not associated with a concomitant decrease in EF. This in accordance with previous observations where enhanced arterial wave reflection was associated with preserved EF but reduced left ventricular function as reflected by ventricular longitudinal strain and tissue imaging (25). Another possible explanation is the fact that earlier arrival of the reflected waves is seen in shorter patients (due to the decreased traveling distance of the waves), and short stature is associated with smaller heart size and volumes; thus, the ratio of the SV to the left ventricular end-diastolic volume remains unchanged. Although left ventricular volumes were not measured in our study, left ventricular mass and end-diastolic diameters were lower in patients, with early reflections suggesting smaller left ventricular cavities (Table 2). Finally, EF was only visually estimated in our study, a method possibly not sensitive enough to detect changes in EF for subtle changes in SV.

Our study highlights the importance of a detailed analysis of the left ventricular afterload for the accurate evaluation of the severity of the AS. Brachial systolic and diastolic pressures are not sufficient since they do not represent the whole spectrum of the mechanical load imposed on the left ventricle. On the other hand, it would be unrealistic to suggest invasive recordings of the aortic pressure for every patient with AS. The use of the handheld, high fidelity tonometers developed in the last years may be an excellent option since they provide accurate, non-invasive measurements of the pressure waveform of an artery close to the skin (26, 27). The subsequent combination of pressure and flow obtained concomitantly during routine echocardiography provides a detailed description of the left ventricular afterload directly at the patient’s bedside.

To attenuate the impact of high after load on TPG, it has been suggested that the assessment of the AS should be repeated after the intravenous or sublingual administration of nitrates (28–34). At conventional dosage, these potent vasodilators act on the wall of the small arteries but have no/little effect on arterioles, large arteries, or the aorta. Since arterioles are unaffected, nitrates do not affect systemic vascular resistance (unless administered in high doses), and their beneficial effect on afterload is considered to be entirely attributable to the reduction in wave reflection amplitude (1). In the absence of a direct effect on large arteries, nitrates do not affect pulse wave velocity, thus have little impact on the delay of the reflected waves (35, 36). Nevertheless, it should be noted that nitrates also have a significant venodilating effect, which results in pooling of the circulating blood volume in the venous circulation and thus a decrease of the blood return to the heart. Since nitrates decrease both preload and afterload, the cumulative effect on SV and aortic flow is not easily predictable and depends on different factors such as the status of the left ventricular function and the presence or not of reflex sympathetic nervous activity (37). Finally, it should be noted that the hemodynamic responses to nitrates may be attenuated by the development of partial or complete nitrate tolerance.

Another important finding of the present study was the association between early reflection wave arrival (Q1) and all cause 1 year mortality. This is in accordance with observations in other populations such as patients with arterial hypertension, end-stage renal disease and coronary artery disease where arterial reflections present a prognostic significance independently of the traditional risk factors (4–7). This may be explained not only by the increased pressure afterload imposed to the left ventricle but also the concomitant decrease in coronary perfusion pressure because of the shift of wave reflections from the diastolic to the systolic period. The cumulative effect of increased afterload and decreased coronary perfusion alters the myocardial oxygen supply-demand ratio and may predispose to ischemia as shown experimentally by Buckberg et al. (38). Interestingly, the prevalence of tricuspid regurgitation (moderate or severe) was also higher in patients with early reflection (Q1), which blunted the prognostic significance of wave reflection in the multivariate Cox-regression model. Further studies are required in order to elucidate the mechanism of this association.

## Limitations

The study is subjected to the limitations of the retrospective, cohort study design. Wave separation analysis was performed by combining flow and pressure data not simultaneously recorded. In case of difference in HR between the pressure and the flow waveforms, synchronization was achieved by truncating or extending the diastolic portion of the flow wave, which may influence accuracy. However, this can only increase the probability of a type II error (false negative, mistaken acceptance of the null hypothesis). Moreover, CO was acquired invasively by two different techniques (thermodilution or the modified Fick method with estimated oxygen consumption) that may not be used interchangeably. Finally, AS classification was performed by the use of invasive SVi estimation, which is not readily available in clinical routine. Thus, the associations with the incidence of low-flow, low gradient severe AS with preserved EF may not apply when SVi is measured by other techniques with higher variability (e.g., TTE Pulsed Wave Doppler).

## Conclusion

Early reflected wave arrival at the aortic root, generated by arterial trees with pronounced impedance mismatch between peripheral and central arteries, is associated with poor prognosis and profound hemodynamic changes at the aortic level including a significant decrease in transvalvular aortic flow and concomitant increase in aortic pressures. This is related to a significant decrease in TPG for a given AVA, leading to the underestimation of the AS. Our study highlights the importance of a detailed analysis of the left ventricular afterload for the accurate evaluation of the AS severity for both diagnostic and prognostic purposes.

## Data Availability Statement

The datasets presented in this article are not readily available because of institutional restrictions applying to data involving human subjects. Requests to access the datasets should be directed to DA, dionysios.adamopoulos@hcuge.ch and SN, stephane.noble@hcuge.ch.

## Ethics Statement

The studies involving human participants were reviewed and approved by Geneva Ethics Committee (Commission Cantonale d’Ethique de la Recherche). The patients/participants provided their written informed consent to participate in this study.

## Author Contributions

SP and DA conceived and designed the research, analyzed the data, prepared the figures, and drafted the manuscript. SN, GG, and HM performed the measurements. SP, NS, and SN interpreted the results. SP, DA, GR, VB, HM, GG, SM-W, M-JL, NS, and SN edited and revised the manuscript and approved the final version of the manuscript.

## Funding

Open access funding was provided by the University of Geneva.

## Conflict of Interest

The authors declare that the research was conducted in the absence of any commercial or financial relationships that could be construed as a potential conflict of interest.

## Publisher’s Note

All claims expressed in this article are solely those of the authors and do not necessarily represent those of their affiliated organizations, or those of the publisher, the editors and the reviewers. Any product that may be evaluated in this article, or claim that may be made by its manufacturer, is not guaranteed or endorsed by the publisher.

## Footnotes

1
www.openclinica.com
