# Erratum: Arterial wave reflection and aortic valve stenosis: Diagnostic challenges and prognostic significance

**DOI:** 10.3389/fcvm.2023.1181506

**Published:** 2023-03-21

**Authors:** Frontiers Production Office

**Affiliations:** Frontiers Media SA, Lausanne, Switzerland

**Keywords:** arterial wave reflection, aortic valve stenosis, arterial stiffness, transvalvular pressure gradients, arterial hypertension

An Erratum on Arterial wave reflection and aortic valve stenosis: diagnostic challenges and prognostic significance by Pagoulatou S, Adamopoulos D, Rovas G, Bikia V, Müller H, Giannakopoulos G, Mauler-Wittwer S, Licker M-J, Stergiopulos N and Noble S. (2022) Front. Cardiovasc. Med. 9:863968. doi: 10.3389/fcvm.2022.863968

An omission to the funding section of the original article was made in error. The following sentence has been added: “Open access funding was provided by the University of Geneva”.

The original version of this article has been updated.

